# Complex interplay between emotional states and gait parameters: a domain-specific investigation in healthy young adults

**DOI:** 10.1007/s00221-025-07048-1

**Published:** 2025-03-24

**Authors:** Bahman Adlou, Danielle Wadsworth, John L. Grace, Jerad Kosek, Christopher Wilburn, Wendi Weimar

**Affiliations:** 1 School of Kinesiology, Auburn University, Auburn, AL USA; 2 School of Health Sciences, University of Evansville, Evansville, IN USA

**Keywords:** Embodied cognition, Motor control, Principal component analysis, Emotional state, Gait

## Abstract

Emotional states significantly influence human movement, yet specific relationships between naturally occurring moods and gait parameters remain poorly understood. This study investigated how naturally occurring mood states affect gait characteristics in young adults (*n* = 16), accounting for time-of-day variations and individual-level differences. Participants completed mood assessments and instrumented gait analysis across five sessions throughout an academic semester. Principal component analysis identified five distinct gait domains (phase, variability, rhythmicity, temporal, and spatial), explaining 84.67% of total variance. Linear mixed-effects models with random intercepts showed that negative moods distinctly affected temporal (β = 0.675, *p* = 0.012) and spatial (β = 0.522, *p* = 0.045) components, with anger and shame prolonging temporal measures and guilt and shame increasing spatial variability. Conversely, happiness shortened temporal measures (β=-0.568) and reduced spatial variability (β=-0.683), suggesting a stabilizing effect on gait dynamics. Substantial inter-individual variability emerged in phase organization (4.870), underscoring unique baseline differences in emotion-gait coupling. Morning sessions exhibited significantly faster gait speed (mean = 1.577 ± 0.296 m/s) and longer step lengths (mean = 80.070 ± 10.767 cm) compared to afternoon sessions (gait speed: mean = 1.311 ± 0.107 m/s; step length: mean = 71.903 ± 5.325 cm; *p* < 0.001). These findings confirm that distinct mood states elicit domain-specific gait changes and highlight the importance of circadian and individual factors for understanding embodied cognition and refining clinical gait assessments.

**Supplementary Information:**

The online version contains supplementary material available at 10.1007/s00221-025-07048-1.

## Introduction

The complex interplay between motor function and emotional processing has become an increasingly significant focus of scientific inquiry. Recent empirical evidence has demonstrated that body movements and postures can substantially influence emotional experiences, and emotional states reciprocally affect motor behavior patterns (Cuddy et al. 2018; Elkjær et al. 2022). This bidirectional relationship aligns with contemporary embodied cognition frameworks, which emphasize that our emotional experiences are intrinsically linked to both physiological states and motor expressions (Winkielman et al. 2015). Seminal work by Troje (2002) established principal component analysis (PCA) as a robust framework for decomposing biological motion into interpretable gait domains (Troje 2002). By isolating kinematic features that distinguish individual walking styles, this approach laid the methodological foundation for investigating emotion-specific gait alterations (Roether et al. 2009). Building on this dimensional reduction approach, Barliya et al. (2013) demonstrated that emotions affect not only walking speed but also modify intersegmental coordination patterns during locomotion. Their analysis of the law of intersegmental coordination revealed that emotional expression produces kinematic alterations that cannot be explained by speed changes alone, with emotions like anger particularly influencing the orientation of the coordination plane between leg segments (Barliya et al. 2013). The integration of emotional and motor systems suggests that our physical manifestations, including both postural configurations and movement patterns, not only reflect but actively contribute to our affective experiences, challenging traditional cognition-centric views of emotion processing.

Walking gait, as a fundamental human movement, serves as an ideal paradigm for investigating emotion-motion relationships. The automaticity of gait, combined with its sensitivity to both cognitive and emotional states, provides a unique window into the embodiment of emotional experiences (Fawver et al. 2015). The work of Hollman et al. (2011) suggests that emotional states can influence various aspects of gait, from basic spatiotemporal parameters to more complex measures of variability and rhythmicity (Hollman et al. 2011).

Additionally, the theoretical framework of approach-avoidance motivation provides a compelling mechanism for understanding how emotions influence gait patterns. Positive emotional states facilitate approach behaviors, characterized by increased gait speed and step length, while negative states lead to withdrawal behaviors reflected in reduced pace and increased cautiousness (Fawver et al. 2015; Lebert et al. 2024). Such two-way relationships manifest across multiple independent domains of gait function, with emotional states differentially affecting spatiotemporal parameters in distinct ways (Homagain and Martens 2023). Previous research has established that emotions may impact pace (characterized by step velocity and length), rhythm (temporal organization of gait cycles), variability (step-to-step fluctuations), asymmetry (differences between limbs), and postural control (balance maintenance during walking) (Lord et al. 2013). Notably, the pace and rhythm domains appear most sensitive to emotional influences, while variability and postural control show less consistent emotional modulation (Homagain and Martens 2023). These emotion-induced alterations in gait emerge during both gait initiation and steady-state walking, suggesting that emotional states shape the entire locomotor process (Fawver et al. 2015; Homagain and Martens 2023).

While laboratory studies have demonstrated robust emotion-motion relationships, most research has relied on artificial emotion induction methods or laboratory-setting gait tests, questioning their ecological validity and potentially limiting generalizability to everyday experiences. Recent studies using virtual reality have improved ecological validity (Kim et al. 2019), but questions remain about how naturally occurring mood fluctuations influence gait patterns throughout daily life. The present study addresses this gap by investigating how self-reported (naturally occurring) mood states relate to gait parameters in young adults. We account for two critical factors: (1) circadian variations, which modulate motor performance via changes in alertness and neuromuscular preparedness, and (2) individual differences, including baseline walking patterns and emotional reactivity. Based on recent evidence and the approach-avoidance framework, we hypothesize that positive mood states will be associated with increased pace domain parameters (step velocity and length), while negative mood states will manifest in reduced pace and altered rhythm and variability parameters. This investigation will advance our understanding of emotion-motion coupling in naturalistic contexts and inform the development of more sensitive gait assessment protocols that account for emotional state and time-of-day testing as a potential confounding factor in both clinical and research settings.

## Methods

### Population

To be eligible for the study, participants had to be enrolled in one of two Tuesday-Thursday sections (morning session at 10am and afternoon session at 2pm) of a core class within the kinesiology curriculum at a large public university (Auburn University, Auburn, USA) and able to walk across an instrumented walkway. Participants were recruited at the beginning of the semester by a member of the research team who was not an instructor for the class. Participants who signed the informed consent and met study eligibility were enrolled in the study (*n* = 16, Table 1). Participants who completed the study were compensated with five bonus points on their final course test. All procedures described herein were approved by the Institutional Review Board and conformed to the standards set by the latest revision of the 1964 Declaration of Helsinki.

**Table 1:** Participant demographics and testing session distribution. †Values are presented as mean ± standard deviation. Data collection occurred over five testing sessions throughout the semester. Three participants completed make-up sessions during their off-week at the same time of the day to replace missing original session dates. Testing compliance varied across participants, with 12 participants completing all five sessions, one participant completing four sessions, and 2 participants completing two sessions

Characteristic	Morning Session	Afternoon Session
*Participants (n = 16)*	*4 (3 females)*	*12 (7 females)*
*Height (cm)†*	*65.6 ± 2.0*	*69.1 ± 3.0*
*Weight (kg)†*	*66.6 ± 7.5*	*77.5 ± 11.5*

### Procedures

Participants were emailed a baseline questionnaire to obtain demographic and anthropometric information, including age, biological sex, gender, self-identified race, height, and weight. Following baseline data collection, participants were randomly assigned to one of five testing times, which were repeated over five successive weeks to complete both mood and gait assessments. On the weeks that they were selected, they were sent an email on Monday evening letting them know that they had been randomly selected for that week. On Tuesday of that same week, participants completed a gait assessment by walking along a predetermined space to measure various spatiotemporal gait characteristics, placed in a quiet space outside of the classroom. Participants then completed the mood assessment survey via an electronic device provided by the research team. This process was repeated each week throughout the semester for a total of five assessments.

Spatiotemporal gait parameters were collected using the OptoGait system (Microgate S.r.l., Italy), consisting of two parallel infrared LED bars (4.9 m length, 1.22 m apart) operating at 1000 Hz. The system detects foot placements via infrared beam interruptions between transmitting (TX) and receiving (RX) bars, measuring anterior-posterior motion with 1.041 cm resolution. Each bar contains 96 LEDs spaced 1.041 cm apart, enabling precise detection of temporal parameters (stance/swing time) and spatial parameters (step length). Participants walked along the 4.9 m instrumented walkway, starting 3 steps before entry to ensure steady-state gait. A key limitation is the system’s restriction to sagittal plane analysis, precluding medio-lateral or vertical movement assessment.

Each participant was asked to walk at self-selected gait speed reflecting their walk to the classroom, without carrying or holding any objects such as backpacks or notebooks. All participants started facing the same direction and were asked to start up to three steps before entering the walkway and take up to three steps beyond the walkway before turning around. Three successful walking gait trials were collected for each participant, ensuring they had completed the entire walkway before resetting to walk again.

Ample space was provided on both ends of the walkway to ensure steady-state gait upon entering and exiting the OptoGait-monitored zone. Data were captured at a sampling frequency of 1000 Hz using OptoGait software (Version 1.13.17.0 Microgate S.r.l, Italy). The software was used for real-time data acquisition and preliminary processing. Walking trials data were initially reduced within the OptoGait software, with the first and last steps of each trial removed to focus on steady-state gait and enhance the reliability of the variables (Kuo and Donelan 2010).

### Measures

#### Mood

Mood was ascertained by assessing current positive and negative affective states. Participants were asked to indicate on a 4-point scale from “not at all” (1) to “completely” (4) how sad, disgusted, angry, guilty, ashamed, frustrated, happy, excited, and alert they were at that moment (Mata et al. 2012). This survey took approximately 2 min. Composite positive affect (Pos-mood) included scores from how happy, excited, and alert participants felt. Composite negative affect (Neg-mood) included scores from how frustrated, sad, disgusted, angry, guilty, and ashamed participants felt. These composite scores were computed by summing individual item responses and then rescaling from 1 to 4 for analytical consistency. Earlier validation of this measure reported strong reliability for both negative (α = 0.92) and positive (α = 0.83) affect factors (Mata et al. 2012). In the current study, we observed acceptable internal consistency for negative affect (α = 0.88) but relatively poor consistency for the positive affect composite (α = 0.52). The variability of responses across different contexts (e.g., academic tasks, morning vs. afternoon sessions) may have dampened the internal coherence of the positive affect dimension.

#### Gait

Gait speed was calculated using the spatiotemporal parameters obtained from the OptoGait system. The calculation utilized step length and step time data to compute gait speed in meters per second (m/s) using the following:

$$\:Gait\:Speed=\frac{\left(Step\:Length*\:Cadence\right)}{60}$$

​where step length is in meters and cadence (steps per minute) is derived from step time. Step length was converted from centimeters to meters, and cadence was calculated as 60 divided by step time in seconds. This method ensures consistent gait speed calculation across all trials and participants.

To account for anthropometric differences and allow for more meaningful inter-individual comparisons, spatial parameters (step length, stride length) were normalized to participant height to account (Hof 1996), and gait speed was further normalized using the dimensionless Froude number approach. The Froude number (Fr) was calculated as:

$$\:Fr=\frac{{v}^{2}}{gL}$$

​where v is gait speed, g is the gravitational constant (9.81 m/s²), and L is leg length. The resulting normalized gait speed values were incorporated into subsequent analyses.

Twenty spatiotemporal parameters were collected during walking trials (Table 2), including temporal parameters (e.g., step time, stance time, swing time), spatial parameters (e.g., step length, stride length), gait speed, phase parameters (e.g., single support, double support percentages), and variability measures (coefficient of variation for each parameter). These variables were selected based on established frameworks for gait analysis in adults (Lord et al. 2013) and validated protocols for comprehensive gait assessment (Hollman et al. 2011).

**Table 2:** Selected gait variables and their definitions. Cv = coefficient of variation; pct = percentage; s = seconds; cm = centimeters. The calculation of the variables by optogait is detailed in supplementary table S1

Suggested Domain	Variable	Definition
*Rhythm*	stepT_s_mean	Mean step time (seconds)
	stanceT_s_mean	Mean stance time (seconds)
	swingT_s_mean	Mean swing time (seconds)
*Pace*	stepL_cm_mean	Mean step length (centimeters)
	strideL_cm_mean	Mean stride length (centimeters)
	gaitSpeed_mean	Mean gait speed (cm/s)
*Variability*	gaitSpeed_cv	Coefficient of variation of gait speed
	stanceT_pct_cv	Coefficient of variation of stance time (% of gait cycle)
	swingT_pct_cv	Coefficient of variation of swing time (% of gait cycle)
	singlesupport_pct_cv	Coefficient of variation of single support time (%)
	doublesupport_pct_cv	Coefficient of variation of double support time (%)
	stanceT_s_cv	Coefficient of variation of stance time (seconds)
	swingT_s_cv	Coefficient of variation of swing time (seconds)
*Asymmetry*	stepT_s_cv	Coefficient of variation of step time (seconds)
	stepL_cm_cv	Coefficient of variation of step length (cm)
	strideL_cm_cv	Coefficient of variation of stride length (cm)
*Phases*	stanceT_pct_mean	Mean stance time (% of gait cycle)
	stanceT_pct_cv	Coefficient of variation of stance time (%)
	swingT_pct_mean	Mean swing time (% of gait cycle)
	swingT_pct_cv	Coefficient of variation of swing time (%)
	singlesupport_pct_mean	Mean single support time (% of gait cycle)
	singlesupport_pct_cv	Coefficient of variation of single support time (%)
	doublesupport_pct_mean	Mean double support time (% of gait cycle)
	doublesupport_pct_cv	Coefficient of variation of double support time (%)

### Statistical analysis

A machine learning algorithm was used to reduce the dimensions of gait without eliminating any meaningful patterns. Principal component analysis with varimax rotation was employed following established frameworks in gait analysis (Lord et al. 2013). Missing values were handled within each participant using median imputation to preserve individual gait characteristics (Aguinis et al. 2013).

The Kaiser-Meyer-Olkin (KMO) test assessed sampling adequacy, with component retention determined through multiple criteria: Kaiser criterion (eigenvalue > 1.0), cumulative variance explained (> 80%), and scree plot examination (Lord et al. 2013a; Verghese et al. 2009). Varimax rotation was applied to enhance component interpretability while maintaining orthogonality (Lord et al. 2013).

Following dimension reduction through PCA with varimax rotation, we employed linear mixed-effects models to examine the relationship between mood states and gait parameters. This approach was selected to account for repeated measures within participants, potential time-of-day effects, and individual variability in gait patterns (Schielzeth et al. 2020). Two separate analyses were conducted: individual mood effects on each rotated component (RC), and composite mood effects (Pos-mood and Neg-mood) on RCs. The mixed-effects models included fixed effects for mood states and session, with random intercepts for participants. Statistical significance was set at α = 0.05. All analyses were performed using Python (v3.12). All analyses were performed using Python (v3.12) with NumPy for numerical computations (Harris et al. 2020), SciPy for statistical analyses (Virtanen et al. 2020), Matplotlib for data visualization (Hunter 2007), and Statsmodels for mixed-effects modeling (Seabold and Perktold 2010).

## Results

Morning session participants were characterized by lower height (*M* = 65.6, *SD* = 2.0 cm) and weight (*M* = 66.6, *SD* = 7.5 kg) compared to afternoon participants (height: *M* = 69.1, *SD* = 3.0 cm; weight: *M* = 77.5, *SD* = 11.5 kg).

### Gait variable dimensionality reduction (KMO & PCA)

**Fig. 1:**
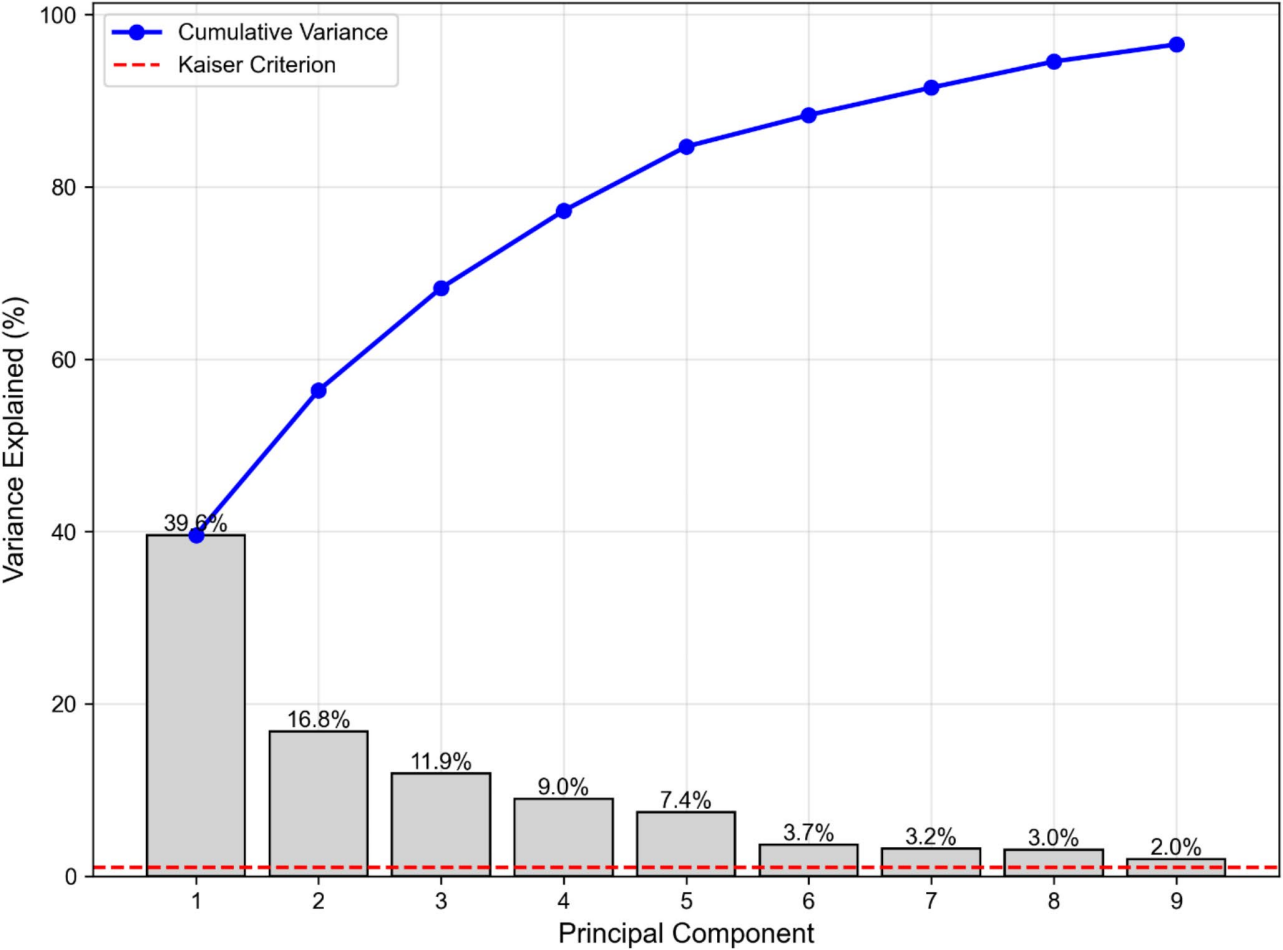
Principal component analysis of gait parameters. PC = Principal Component. The bar graph shows individual variance explained by each principal component (gray bars) and cumulative variance (blue line). The red dashed line represents the Kaiser criterion (eigenvalue > 1.0). The first five components explained 84.67% of total variance, with individual contributions of PC1 (39.57%), PC2 (16.80%), PC3 (11.89%), PC4 (8.96%), and PC5 (7.45%). Nine components with eigenvalues above 1.0 were extracted, explaining 96.53% of total variance, though only five components were retained based on theoretical framework and interpretability

The KMO measure (0.799) indicated meritorious sampling adequacy (Kaiser 1974). Nine components with eigenvalues > 1.0 were extracted, explaining 96.53% of total variance (Fig. 1). The rotated component solution revealed five distinct gait domains, explaining 84.67% of total variance, aligning closely with the five-factor model established by Lord et al. (2013a, b)(Lord, Galna, Verghese, et al., 2013).

The rotated solution provided clearer separation of gait domains through five primary components (complete comparison between original and varimax-rotated loadings is provided in Supplementary Figure S1). RC1 (Phase Component) explained the temporal organization of the gait cycle, with significant negative loadings for stance time (-0.422) and double support time (-0.424), balanced by positive loadings for swing phase (0.421) and single support (0.411) parameters. RC2 (Variability Component) captured stability aspects of gait, dominated by double support time variability (0.786) and single support variability (0.492). RC3 (Rhythmicity Component) reflected the consistency of stepping patterns through stance (0.565) and swing time (0.585) variability measures. RC4 (Temporal Component) represented basic timing of gait cycle with strong loadings for swing time (0.704) and step time (0.551). RC5 (Spatial Component) primarily captured spatial aspects and asymmetry, with high loading for step length variability (0.843) and swing time variability (0.412). The complete component structure is presented in Table 3.

**Table 3:** Rotated component matrix of gait parameters. RC = Rotated component. Values represent component loadings after varimax rotation. Only loadings ≥ 0.3 are displayed. Empty cells indicate loadings < 0.3. component labels: RC1 (Phase), RC2 (Variability), RC3 (Rhythmicity), RC4 (Temporal), and RC5 (Spatial)

Variable	RC1	RC2	RC3	RC4	RC5
stepT_s_mean				0.551	
stepT_s_cv					
stepL_cm_mean					
stepL_cm_cv					0.843
strideL_cm_mean					
strideL_cm_cv					
gaitSpeed_mean					
gaitSpeed_cv					
stanceT_pct_mean	-0.422				
stanceT_pct_cv			0.565		
swingT_pct_mean	0.421				
swingT_pct_cv			0.585		
singlesupport_pct_mean	0.411				
singlesupport_pct_cv		0.492			
doublesupport_pct_mean	-0.424				
doublesupport_pct_cv		0.786			
stanceT_s_mean	-0.315			0.388	
stanceT_s_cv			0.554		
swingT_s_mean				0.704	
swingT_s_cv					0.412

**Fig. 2:**
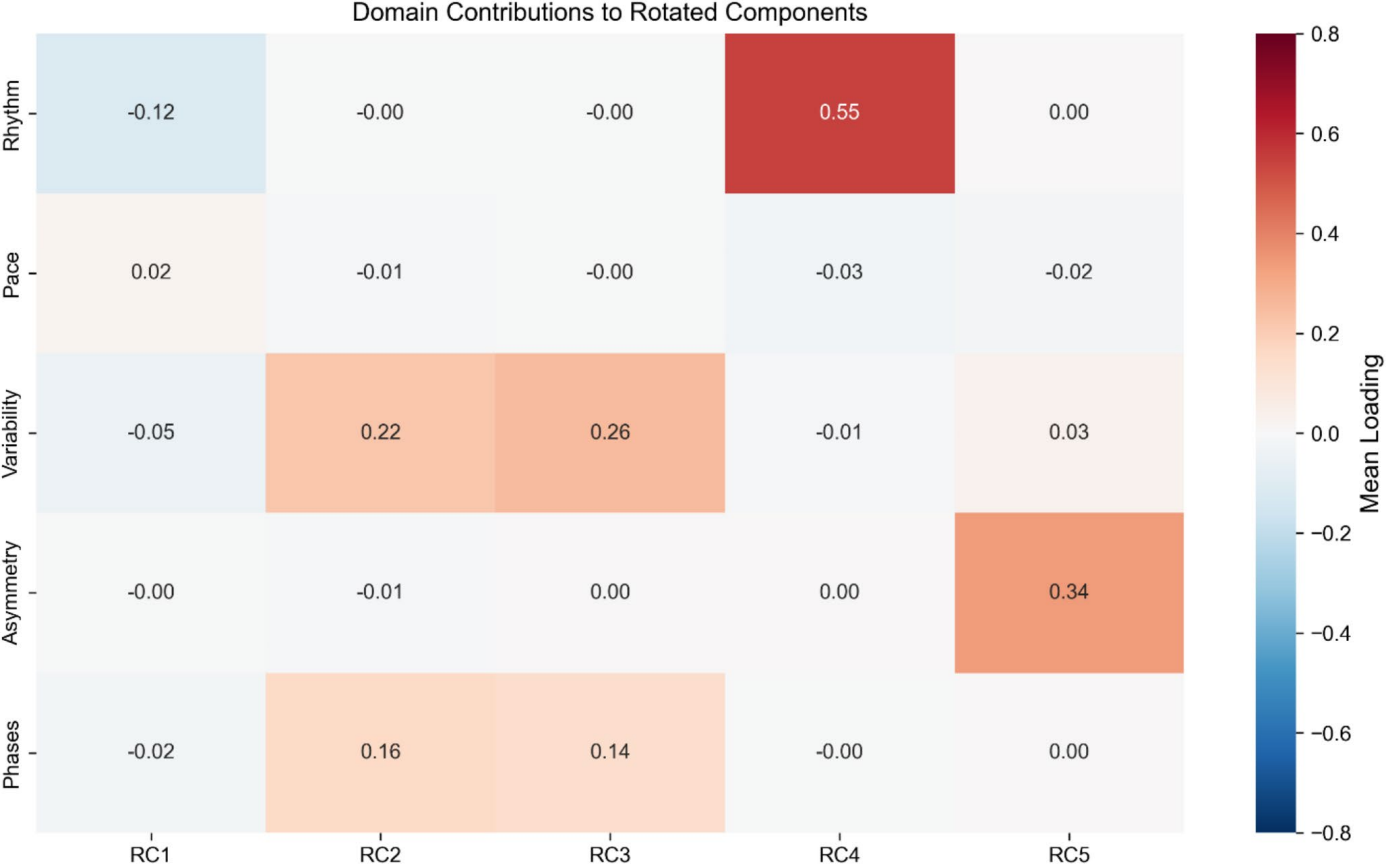
Domain Contributions to Rotated Components. Heat map visualization of mean contributions from each gait domain (Rhythm, Pace, Variability, Asymmetry, Phases) to the five rotated components (RC1-RC5). Color intensity represents the strength and direction of domain contributions, with darker red indicating stronger positive contributions and darker blue indicating stronger negative contributions. The distinct loading patterns validate the five-component structure and support its utility for comprehensive gait assessment

Figure 2 illustrates the mean contributions of gait domains to each rotated component, demonstrating distinct loading patterns across components.

### Preliminary analysis

Mood assessment revealed a higher positive affect score across all participants (*M* = 2.98, *SD* = 0.51, range: 1.67-4.00) compared to negative affect scores (*M* = 1.53, *SD* = 0.52, range: 1.00-3.20). Mood scores showed minimal variation between testing sessions. Participants in morning sessions reported positive affect scores averaging 3.00 (SD = 0.64) on a 1–4 scale, closely matching afternoon participants’ scores (*M* = 2.97, *SD* = 0.46). Negative affect scores were similarly comparable between groups, with morning participants averaging 1.43 (*SD* = 0.65) and afternoon participants 1.56 (*SD* = 0.47).

Analysis of gait parameters revealed significant session-dependent differences in multiple domains. Morning session participants demonstrated faster gait speed (*M* = 1.577, *SD* = 0.296 m/s) compared to afternoon participants (*M* = 1.311, *SD* = 0.107 m/s; *p* < 0.001, d = 1.490). This was accompanied by longer step lengths in the morning session (*M* = 80.070, *SD* = 10.767 cm vs. *M* = 71.903, *SD* = 5.325 cm; *p* = 0.006, d = 1.135) and corresponding stride length differences (*M* = 160.113, *SD* = 21.357 cm vs. *M* = 143.700, *SD* = 10.715 cm; *p* = 0.004, d = 1.143).

Temporal parameters showed shorter step times in the morning session (*M* = 0.513, *SD* = 0.038 s vs. *M* = 0.549, *SD* = 0.031 s; *p* = 0.001, d = -1.086), with corresponding differences in stance time (*M* = 0.623, *SD* = 0.074 s vs. *M* = 0.689, *SD* = 0.043 s for morning and afternoon sessions, respectively; *p* = 0.001, d = -1.240).

Phase distribution analysis revealed that morning participants spent proportionally less time in stance phase (*M* = 60.743, *SD* = 2.916% vs. *M* = 62.819, *SD* = 1.420% for morning and afternoon, respectively; *p* = 0.002, d = -1.072) and double support (*M* = 22.047, *SD* = 5.440% vs. *M* = 25.847, *SD* = 2.732% for morning and afternoon, respectively; *p* = 0.002, d = -1.039), with correspondingly higher proportions of swing phase (*M* = 39.243, *SD* = 2.887% vs. *M* = 37.181, *SD* = 1.420% for morning and afternoon, respectively; *p* = 0.002, d = 1.071) and single support (*M* = 39.038, *SD* = 2.766% vs. *M* = 37.141, *SD* = 1.387% for morning and afternoon, respectively; *p* = 0.002, d = 1.020).

The magnitude of these differences was substantial, with large effect sizes (Cohen’s|d| > 0.8) observed across several parameters, particularly in speed-related measures and temporal phase distributions. Variability measures showed less consistent patterns between sessions, with significant differences observed only in single support variability (*p* = 0.008) and swing time variability (*p* = 0.018). Collectively, these findings indicate that morning participants exhibited a more dynamic gait pattern characterized by faster walking speed, longer steps, and reduced time spent in double support, suggesting enhanced gait stability and efficiency compared to afternoon participants.

### Mood effects on gait components

The mixed-effects analysis revealed significant associations between specific mood states and gait parameters, as represented by the rotated components (RCs). To account for individual-level differences, we included random intercepts for participants in our linear mixed-effects models, enabling each individual to have a unique baseline gait profile (Schielzeth et al. 2020). The random intercept variance (Var(RE)) quantifies between-participant variability, while the residual variance (Var(Residual)) captures within-participant fluctuations. Together with the fixed effects, these components contribute to the total explained variance (Var(Explained)) in each rotated component.

The random intercept variance ranged from 0.109 to 5.571 across the five RCs (see Supplementary Table S2), highlighting the considerable influence of participant-level differences on gait parameters. In RC1 (Phase), Var(RE) was notably high (4.870), indicating substantial inter-individual variability in gait-phase organization; this component also had a relatively large Var(Explained) value (0.814), signifying that mood states and participant-level factors jointly accounted for most of the variance in phase-related measures. By contrast, RC5 (Spatial) exhibited comparatively lower values of Var(RE) (0.109–0.180), meaning less between-participant variability in spatial parameters, and modest Var(Explained) (0.078–0.125), implying that unmeasured influences beyond mood and baseline gait differences likely shape spatial variability.

**Fig. 3:**
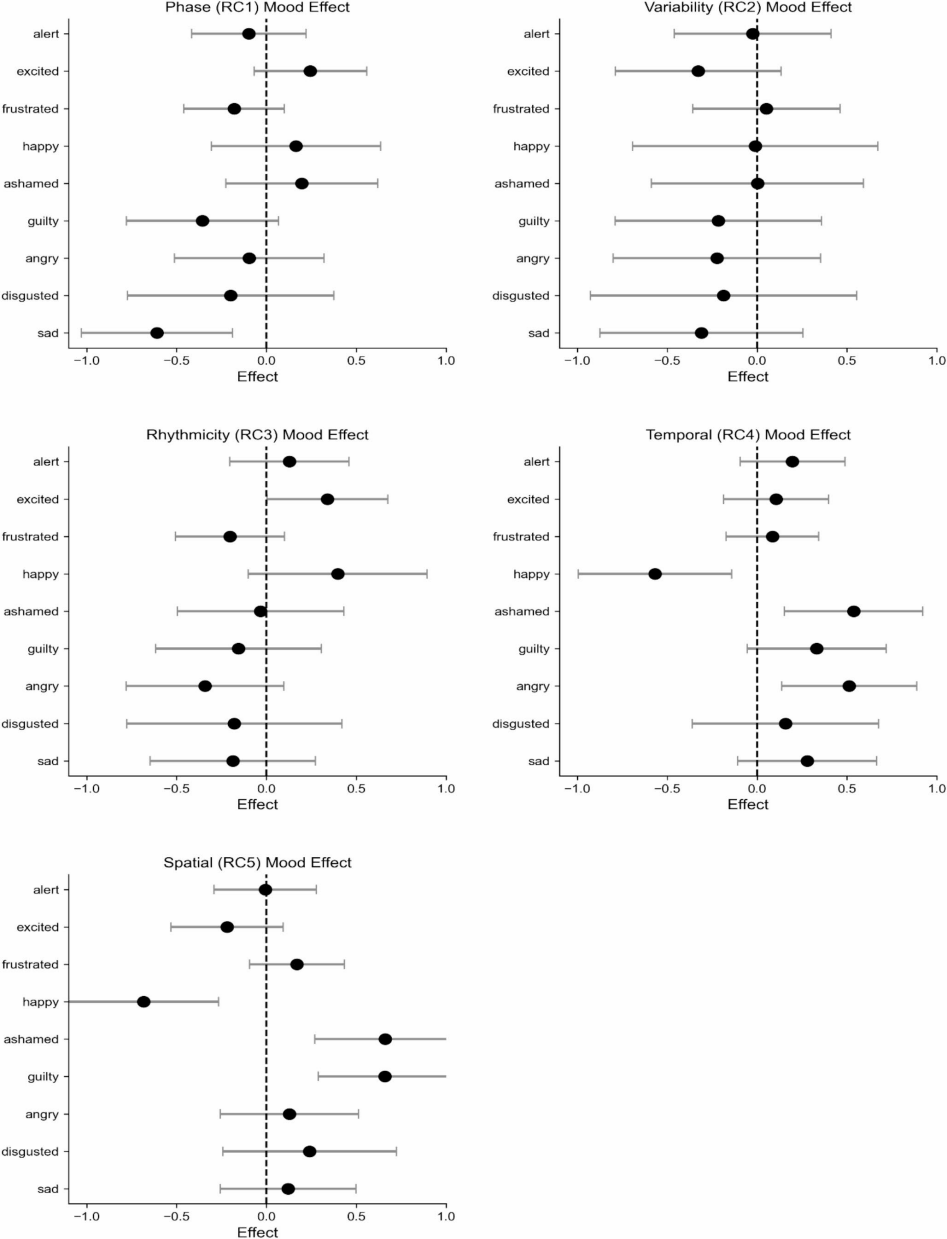
Mood effects on gait components. Forest plot showing standardized effect sizes (β) and 95% confidence intervals for significant mood-gait associations. Negative values indicate inverse relationships

Individual mood effects showed distinct patterns across components. Phase component parameters demonstrated a significant negative association with sadness (β = -0.609, *p* = 0.005), indicating that increased sadness was associated with altered temporal organization of gait. Rhythmicity showed a positive relationship with excitement (β = 0.339, *p* = 0.047), suggesting that higher excitement levels were associated with more consistent (*rhythmic*) stepping patterns. The most pronounced effects were observed in Temporal and Spatial components, where multiple mood states showed significant associations (Fig. 3; further detailed statistics provided in Supplementary Table S2). In the Temporal domain of gait, anger (β = 0.512, *p* = 0.007) and shame (β = 0.537, *p* = 0.006) demonstrated positive associations, while happiness showed a negative relationship (β = -0.568, *p* = 0.009). Similar patterns were observed in Spatial domain, with guilt (β = 0.659, *p* < 0.001) and shame (β = 0.660, *p* = 0.001) showing positive associations, and happiness showing a negative relationship (β = -0.683, *p* = 0.001).

Analysis of composite mood effects revealed significant associations primarily in the later components (Table 4). Neg-mood showed significant relationships with both Temporal (β = 0.675, *p* = 0.012) and Spatial components (β = 0.522, *p* = 0.045), while Pos-mood demonstrated a trending but non-significant association with Rhythmicity component of walking gait (β = 0.488, *p* = 0.068).

**Table 4:** Associations between composite affect and gait components. RC = Rotated component; CI = confidence interval. *Significant associations (*p* < 0.05) are observed for negative affect in RC4 and RC5

Component	Affect Type	Effect Size	Standard Error	*p*-value	95% CI
Phase RC1	Positive	0.124	0.983	0.621	-0.368, 0.616
	Negative	-0.339	1.185	0.262	-0.931, 0.254
Variability RC2	Positive	-0.332	1.428	0.362	-1.047, 0.382
	Negative	-0.356	1.571	0.374	-1.142, 0.429
Rhythmicity RC3	Positive	0.488	1.047	0.068	-0.036, 1.012
	Negative	-0.197	1.201	0.521	-0.797, 0.404
Temporal RC4	Positive	0.137	0.911	0.555	-0.318, 0.593
	Negative	0.675	1.055	0.012*	0.148, 1.202
Spatial RC5	Positive	-0.346	0.942	0.150	-0.817, 0.125
	Negative	0.522	1.023	0.045*	0.011, 1.033

When mood was aggregated into positive (Pos-mood) and negative (Neg-mood) categories, RC1 (Phase) showed high explained variance (0.830) but nonsignificant effects for both Pos-mood (β = 0.124, *p* = 0.621) and Neg-mood (β = -0.339, *p* = 0.262). RC4 (Temporal) and RC5 (Spatial) were more sensitive to composite negative affect, with Neg-mood exhibiting significant relationships in RC4 (β = 0.675, *p* = 0.012) and RC5 (β = 0.522, *p* = 0.045). These composite findings further emphasize that negative affect broadly influences the temporal and spatial dimensions of gait, whereas positive affect showed only a trending relationship with RC3 (β = 0.488, *p* = 0.068).

## Discussion

This study aimed to examine how naturally occurring mood states relate to domain-specific gait parameters in college-aged adults, incorporating both time-of-day variations and individual-level differences. Results demonstrated domain-specific relationships between mood states and gait parameters, strengthening evidence that emotional states exert nuanced influences on locomotion rather than inducing uniform changes across all gait domains. Building on the approach-avoidance framework, our findings suggest that negative emotions such as anger, guilt, and shame not only diminish the pace of walking but also alter the spatial variability of each stride, supporting the notion that threat-related affect is coupled with more cautious and variable gait patterns (Lebert et al. 2024). By contrast, positive states such as happiness appear to shift gait toward more dynamic and expansive movement. Furthermore, substantial participant-level variance highlighted the importance of individual differences in shaping emotion-gait interactions, while the time-of-day findings may underscore chronobiological considerations relevant to gait assessment.

Recent machine learning advances have refined principal component analysis utility for emotion recognition. For example, ensemble models could classify sadness via step-length variability (Xu et al. 2024). Our findings extend this work by demonstrating that PCA-derived domains remain sensitive to naturally occurring moods outside laboratory settings, suggesting machine learning approaches could enhance ecological emotion-gait modeling while preserving interpretability.

The PCA in this study yielded five distinct gait domains (phase, variability, rhythmicity, temporal, and spatial), explaining 84.67% of the variance consistent with established frameworks in gait research (Lord et al. 2013; Lord, Galna, Verghese, Lord et al. 2013a, b). This structural similarity validates our analytical approach and confirms that fundamental gait organization principles remain consistent even in a young adult population. Although prior research suggests that the structural principles of gait remain consistent across age groups (Homagain and Martens 2023), our findings highlight that distinct emotions may preferentially alter timing or spatial aspects of walking in younger adults. Such granularity extends previous research demonstrating that sadness reduces gait speed (Homagain and Martens 2023), thereby illustrating that different negative emotions can exert unique effects on specific gait domains rather than uniformly slowing or destabilizing locomotion (Boudarham et al. 2013).

When examining composite affect scores, negative mood (Neg-mood) significantly influenced both temporal (β = 0.675) and spatial (β = 0.522) parameters, whereas positive mood (Pos-mood) showed only a marginal relationship with rhythmicity. These findings align with Barliya et al. (2013), who demonstrated that different emotions distinctly affect locomotion kinematics through changes in the amplitudes of elevation angles and intersegmental coordination patterns (Barliya et al. 2013). Their observation that emotional expression causes modifications to locomotion patterns beyond speed-related changes supports our domain-specific findings, particularly regarding the differential effects of discrete emotions on temporal and spatial parameters. This evidence further underscores the need to distinguish between discrete negative emotions rather than treating them as a singular construct, as anger and shame demonstrated varying strengths of association with temporal and spatial domains. Such specificity is supported by the proposed differentiations among negative affect states, where anger may evoke heightened arousal and more forceful motor output, while shame might trigger avoidance tendencies through careful and hesitant movements (Winkielman et al. 2015). Thus, analyzing discrete emotional states may illuminate the nuanced ways in which affect interacts with gait, an approach that could be especially valuable when extending this work to clinical populations experiencing prolonged affective disturbances. These data collectively advance understanding of how gait could act as both an expression of, and a potential mechanism for, regulating emotion.

A key methodological advance in this work was the inclusion of random intercepts in mixed-effects models, which revealed substantial participant-level variance in selected domains (e.g., RC1: Phase, Var(RE) = 4.870). This underscores the role of inter-individual differences—potentially encompassing habitual walking styles, nutrition, sleeping patterns, physiological traits, or emotional coping strategies in shaping emotion-gait relationships. For example, participants with higher baseline variability or reactivity may exhibit amplified mood effects on gait, suggesting the need for targeted interventions in clinical contexts (Hollman et al. 2011). Future research evaluating specific individual difference variables (e.g., personality traits, habitual physical activity patterns, nutrition, or physiological arousal levels) could help elucidate which subgroups are most susceptible to mood-related gait alterations. By identifying these moderators, researchers and clinicians could better predict how emotional states influence locomotion and tailor interventions to account for individual variability.

Time of day emerged as an additional moderating factor, with morning participants demonstrating significantly faster gait speeds (*M* = 1.577, *SD* = 0.296 m/s vs. *M* = 1.311, *SD* = 0.107 m/s), longer step lengths (*M* = 80.070, *SD* = 10.767 cm vs. *M* = 71.903, *SD* = 5.325 cm), and less time in stance and double support phases than afternoon participants. Interestingly, these results diverge from some prior research suggesting that motor performance peaks during late afternoon or evening hours, when core body temperature and alertness may be elevated (Bessot et al. 2020; Vaz et al. 2023). One possibility is that the schedules and routines of young adults in our sample prompt greater neuromuscular readiness during morning sessions, especially if participants typically attend lectures around that time, whereas cognitive load or fatigue may accumulate throughout the day. Alternatively, individual differences in chronotype (i.e., whether one is a ‘morning person’ or an ‘evening person’) could explain why specific walking performance was observed in the morning for this cohort. These findings suggest that time of day should be carefully considered and documented when investigating mood-gait interactions, particularly in populations with variable daily routines such as college students.

### Limitations

Several methodological considerations warrant attention in interpreting our findings. While our sample size (*n* = 16) aligns with similar gait studies in young adults, it limits broader generalizability, particularly regarding time-of-day effects due to uneven distribution between morning and afternoon sessions. Participants self-selected into morning or afternoon course sections based on personal schedules, academic preferences, or chronotype tendencies (e.g., “morning person”), potentially introducing confounding factors such as pre-existing circadian adaptations or lifestyle habits that could independently influence gait patterns. The poor internal consistency for positive affect (α = 0.52) compared to negative affect (α = 0.88) suggests potential measurement limitations in capturing positive emotional states. Moreover, while assessments were repeated five times, mood and gait were measured sequentially at each session, limiting inferences regarding the temporal ordering of emotion and movement changes. Additionally, single time-point mood assessments may not fully capture the dynamic nature of emotional states throughout the day. While the OptoGait system’s sagittal-plane analysis limited multi-dimensional assessment, its high temporal resolution (1000 Hz) and ecological validity enabled capture of mood-gait interactions relevant to our PCA framework (84.67% variance explained). Future studies could integrate 3D inertial sensors and sophisticated motion capture to explore multiplanar effects without sacrificing naturalistic contexts. Furthermore, the chronobiological differences observed may be particularly pronounced in this population due to unique schedules and routines. Future studies could adopt continuous mood monitoring (e.g., ecological momentary assessment), expand to more diverse populations, and probe specific individual difference variables, such as personality traits, habitual physical activity, nutrition, or physiological arousal, that may mediate or moderate the emotion-gait linkage.

In summary, this study provides compelling evidence that discrete emotions and composite mood states can produce domain-specific alterations in gait, a phenomenon further shaped by individual baseline characteristics and chronobiological factors. Negative emotions selectively prolonged temporal variables or heightened spatial variability, whereas happiness conversely shortened temporal metrics and reduced spatial fluctuations. The observed time-of-day effects corroborate the notion that circadian phenomena may moderate emotion-motor interactions. Collectively, these insights advance our understanding of how naturally occurring moods and core gait processes are associated and emphasize the importance of considering participant-level variance and testing times in both research and clinical practice. Future research employing longitudinal paradigms, larger sample sizes, individual characteristics, volitional health behaviors, and continuous mood monitoring will help illuminate the dynamic nature of emotion-gait coupling and its broader implications for health and mobility.

## Electronic supplementary material

Below is the link to the electronic supplementary material.

Supplementary Material 1

## Data Availability

The research data that support the findings of this study have been deposited in Figshare and can be accessed here: 10.6084/m9.figshare.28559201.v2.
