# Supplementary material for: Complex interplay between emotional states and gait parameters: a domain-specific investigation in healthy young adults
Source: Exp Brain Res. 2025 Mar 24;243(4):100. doi: 10.1007/s00221-025-07048-1 (PMC11933162; doi:10.1007/s00221-025-07048-1)
Supplement: Supplementary file 1 — Supplementary Material 1 [file 221_2025_7048_MOESM1_ESM.docx]

# Appendix:

Supplementary Table S1: Gait Variable Definitions and Calculations. Coefficient of variation (cv) is calculated as the standard deviation divided by the mean, expressed as a percentage. Mean values (_mean) represent the arithmetic average of the measure across all steps during the trial. All temporal parameters are measured in seconds (s) or as a percentage of the gait cycle (pct). Spatial parameters are measured in centimeters (cm).

| Variable | Calculation Description |
| --- | --- |
| doublesupport_pct_cv | Coefficient of variation of the sum of two partial double supports (DS1 + DS2), expressed as percentage of gait cycle |
| doublesupport_pct_mean | Average of the sum of two partial double supports (DS1 + DS2), expressed as percentage of gait cycle |
| gaitSpeed_cv | Coefficient of variation of the average speed calculated between two consecutive steps |
| gaitSpeed_mean | Average speed calculated between two consecutive steps |
| singlesupport_pct_cv | Coefficient of variation of the time between last contact of current support and first contact of following support of same foot, expressed as percentage of gait cycle |
| singlesupport_pct_mean | Average time between last contact of current support and first contact of following support of same foot, expressed as percentage of gait cycle |
| stanceT_pct_cv | Coefficient of variation of the time between first and last contact of two consecutive supports of same foot, expressed as percentage of gait cycle |
| stanceT_pct_mean | Average time between first and last contact of two consecutive supports of same foot, expressed as percentage of gait cycle |
| stanceT_s_cv | Coefficient of variation of the time between first and last contact of two consecutive supports of same foot, expressed in seconds |
| stanceT_s_mean | Average time between first and last contact of two consecutive supports of same foot, expressed in seconds |
| stepL_cm_cv | Coefficient of variation of the distance between the heel contact point of one foot to the heel contact point of the opposite foot |
| stepL_cm_mean | Average distance between the heel contact point of one foot to the heel contact point of the opposite foot |
| stepT_s_cv | Coefficient of variation of the time between first contact of a foot and first contact of opposite foot |
| stepT_s_mean | Average time between first contact of a foot and first contact of opposite foot |
| strideL_cm_cv | Coefficient of variation of the distance between two consecutive heel contact points of the same foot |
| strideL_cm_mean | Average distance between two consecutive heel contact points of the same foot |
| swingT_pct_cv | Coefficient of variation of the time between last contact of first support and first contact of following support, expressed as percentage of gait cycle |
| swingT_pct_mean | Average time between last contact of first support and first contact of following support, expressed as percentage of gait cycle |
| swingT_s_cv | Coefficient of variation of the time between last contact of first support and first contact of following support, expressed in seconds |
| swingT_s_mean | Average time between last contact of first support and first contact of following support, expressed in seconds |


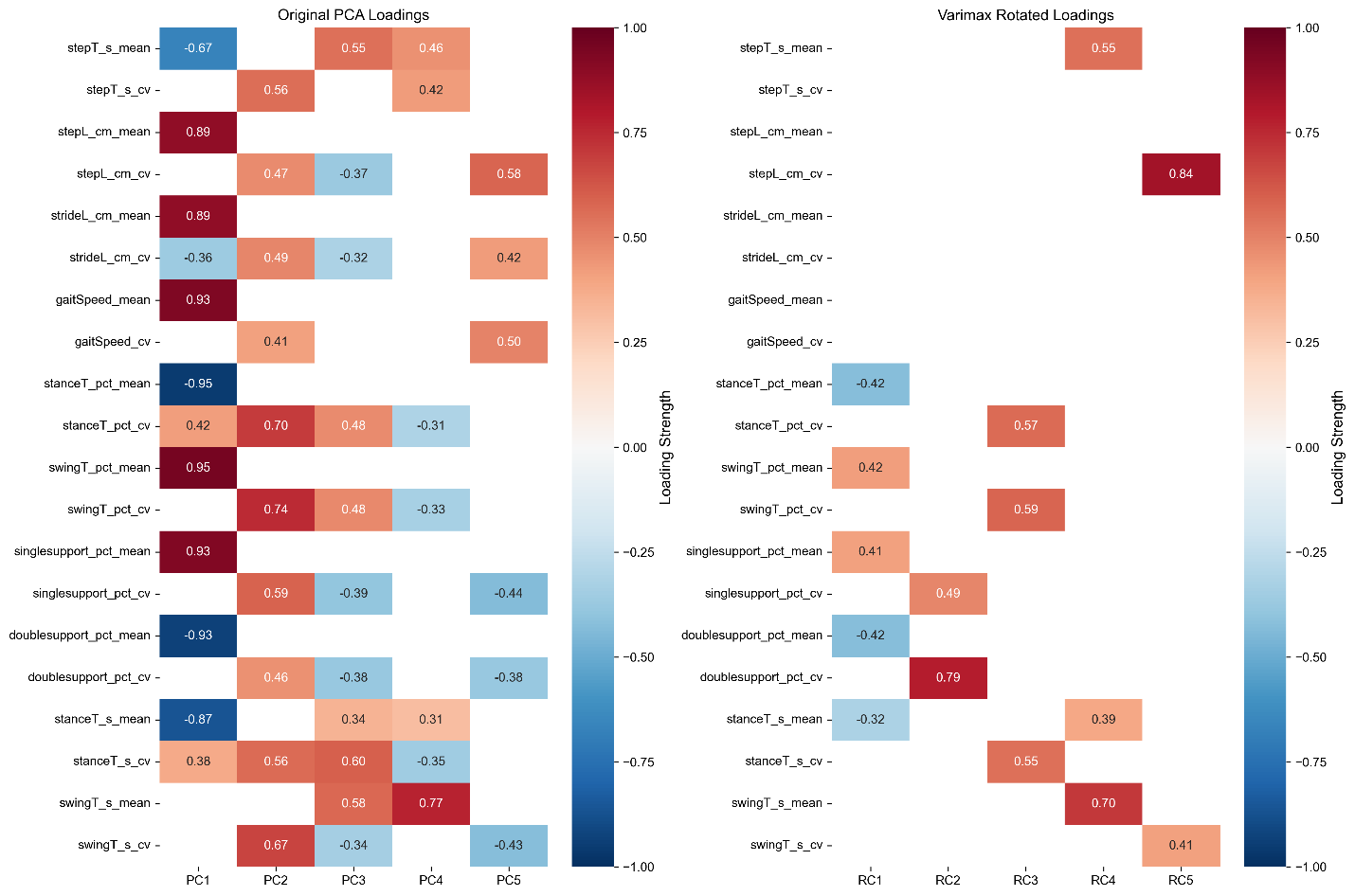


**Supplementary Fig 1.** Comparison of Original and Varimax-Rotated PCA Loadings. Heat maps showing loading patterns before (left) and after (right) varimax rotation. Color intensity represents loading magnitude, with red indicating positive and blue indicating negative loadings. The rotation procedure enhanced component interpretability by maximizing loading separation across variables.

Supplementary Table S2. Associations Between Mood States and Gait Components. Only statistically significant associations (p < 0.05) are shown. Effect sizes represent standardized coefficients (β) from the mixed-effects model. Participant specific variance, residual variance, and variance explained by both the random intercept (participants) and fixed effects are provided in the table. CI = Confidence Interval; RC = Rotated Component.

| Rotated Component | Mood State | Effect Size | Standard Error | p-value | 95% CI | Variability by participant | Residual variance | Explained variance |
| --- | --- | --- | --- | --- | --- | --- | --- | --- |
| Phase (RC1) | Sad | -0.609 | 0.842 | 0.005 | -1.030, -0.188 | 4.870 | 1.112 | 0.814 |
| Rhythmicity (RC3) | Excited | 0.339 | 0.670 | 0.047 | 0.004, 0.675 | 1.083 | 1.342 | 0.447 |
| Temporal (RC4) | Angry | 0.512 | 0.750 | 0.007 | 0.137, 0.887 | 1.035 | 0.978 | 0.514 |
|  | Ashamed | 0.537 | 0.772 | 0.006 | 0.151, 0.922 | 0.893 | 1.000 | 0.496 |
|  | Happy | -0.568 | 0.852 | 0.009 | -0.994, -0.142 | 1.007 | 0.996 | 0.503 |
| Spatial (RC5) | Guilty | 0.659 | 0.741 | <0.001 | 0.289, 1.029 | 0.137 | 1.258 | 0.098 |
|  | Ashamed | 0.660 | 0.783 | 0.001 | 0.269, 1.052 | .0180 | 1.252 | 0.125 |
|  | Happy | -0.683 | 0.835 | 0.001 | -1.100, -0.265 | 0.109 | 1.285 | 0.078 |


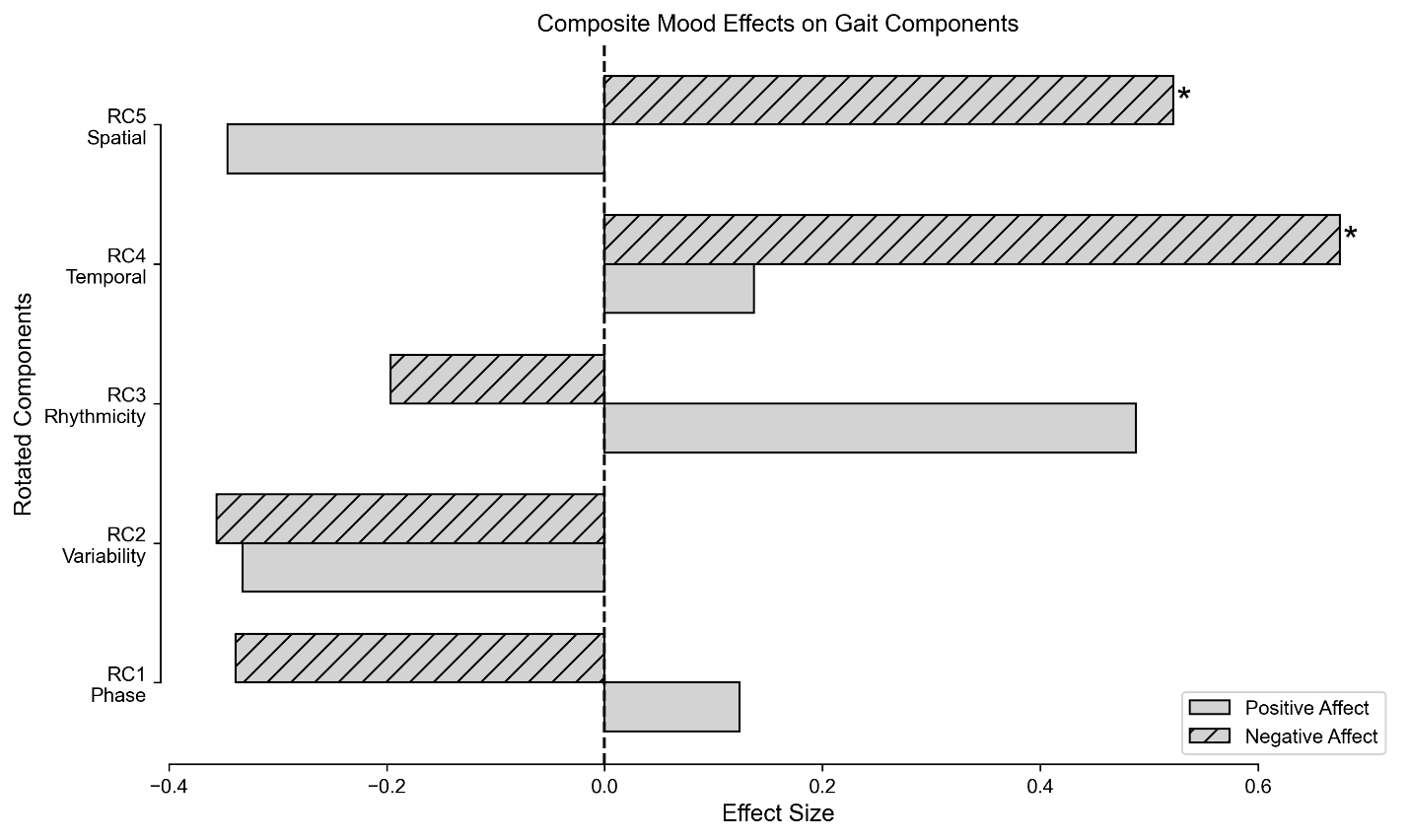


**Supplementary Fig 2**. Composite Mood Effects on Gait Components. Bar plot showing the effect sizes of positive and negative affect on each rotated component (RC) of gait. RC1 represents the Phase component, RC2 Variability, RC3 Rhythmicity, RC4 Temporal, and RC5 Spatial aspects of gait. Gray bars indicate positive affect, while striped bars represent negative affect. Asterisks (*) denote statistically significant effects (p < 0.05). The x-axis shows the standardized effect size, with negative values indicating inverse relationships. This visualization demonstrates the differential impacts of positive and negative mood states across various dimensions of gait, with notable significant effects observed in the Temporal (RC4) and Spatial (RC5) components.
